# Teachers’ Perceptions of Shared Leadership and Their Relationship with Organizational Attractiveness and Identification: A Structural Equation Modeling Approach

**DOI:** 10.3390/jintelligence13110141

**Published:** 2025-11-05

**Authors:** Nesip Demirbilek

**Affiliations:** Genç Vocational School, Bingöl University, 12500 Bingöl, Türkiye; ndemirbilek@bingol.edu.tr

**Keywords:** shared leadership, organizational attractiveness, organizational identification, structural equation modeling

## Abstract

This study examined the relationships among shared leadership, organizational attractiveness, and organizational identification among teachers in Malatya, Türkiye. Using a relational design and structural equation modeling, the research explored how these variables interact. Data were collected via validated scales, revealing positive and significant associations among shared leadership, organizational attractiveness, and organizational identification. Shared leadership and organizational identification were found to significantly predict organizational attractiveness, explaining a substantial portion of its variance. The model demonstrated a good fit, supporting theoretical frameworks related to social identity and shared leadership. Findings highlight that participative leadership is positively associated with teachers’ perceptions of their organizations within a centralized education system. This study offers valuable implications for school leaders and policymakers seeking to enhance teacher engagement and organizational culture. Limitations include the study’s regional focus and cross-sectional design, underscoring the need for broader, longitudinal research to deepen understanding in diverse contexts.

## 1. Introduction

Educational institutions in Türkiye operate within a highly centralized and hierarchical system governed by the Ministry of National Education (MoNE), which oversees curriculum design, teacher appointments, and resource allocation across all public schools (Eurydice 2024). This structure ensures uniformity but often limits school-level autonomy, with decisions on leadership and administration typically flowing top-down from provincial directorates to individual schools (OECD 2023). Despite recent reforms, such as the 2024 “Century of Türkiye Education Model,” which introduces elements of contextual planning and teacher reflection to enhance adaptability (MoNE 2024), the system remains predominantly centralized, with low flexibility for localized decision-making. Teachers face a substantial workload, averaging 24–30 teaching hours per week plus administrative duties, exacerbated by regional inequalities (e.g., urban vs. rural resource disparities) and a teacher-to-student ratio of approximately 1:18 in primary schools (UNESCO 2023). This context amplifies the importance of shared leadership, as it counters hierarchical rigidity by promoting teacher involvement, potentially associated with improved organizational attractiveness and identification in diverse school types (public, private, vocational).

Educational institutions function not only as entities that contribute to individuals’ cognitive development but also as fundamental social organizations that enable individuals to adopt social roles, internalize cultural values, and participate in economic capital production. This multifaceted functionality positions educational institutions as some of the most strategic institutions in modern societies. The effective fulfillment of these roles largely depends on teachers’ professional commitment, level of organizational identification, and participation in administrative processes (Leithwood et al. 2020; Özsarı and Kara 2024).

The uncertainties, diversity, and dynamic changes faced by education systems today have exposed the inadequacies of traditional, centralized, and single-agent leadership models. Especially in school organizations, where decision-making and responsibility-sharing have become multi-actor processes, leadership must be distributed horizontally, requiring active teacher involvement in these processes. In this context, shared leadership has emerged as a prominent concept in recent educational management literature (Harris 2004; Katıtaş et al. 2025; Spillane 2006).

Shared leadership is a collective, interaction-based, and participatory leadership approach in which leadership roles are assumed not only by school principals or administrators but also collectively among teachers and other school stakeholders (Pearce and Conger 2003). This leadership style is associated with trust-based relationships among individuals, contributing to the development of a collaborative culture within school organizations (Carson et al. 2007). It also promotes teachers’ active participation in decision-making processes, positively associated with their professional motivation, organizational commitment, and job satisfaction (Hulpia et al. 2011; Printy and Marks 2006; Kantos et al. 2023). Shared leadership differs from other leadership models, such as transformational leadership, which emphasizes individual leader charisma and vision, by focusing on collaborative influence and mutual accountability among team members (Hoch 2013). Recent studies have further demonstrated its relevance in educational settings, showing that shared leadership enhances teacher self-efficacy and job satisfaction by fostering a sense of ownership and collective responsibility (Katıtaş et al. 2025; Özdemir et al. 2025). Moreover, international research highlights the role of emotional dynamics in leadership, with Paganin et al. (2023) demonstrating that emotional contagion among teachers mediates the relationship between leadership practices and team cohesion, suggesting shared leadership’s broader psychological impact in educational settings.

The applicability of shared leadership in centralized education systems, such as Türkiye’s, is particularly significant. In such systems, where hierarchical structures limit teacher autonomy, shared leadership can empower educators by redistributing decision-making responsibilities, thereby mitigating the constraints of top-down governance (Özer and Beycioğlu 2013). For instance, Özdemir et al. (2025) found that shared leadership in Turkish schools is positively associated with academic optimism and meaningful work, suggesting its potential to transform organizational dynamics even in rigid systems. Moreover, shared leadership aligns with social identity theory by fostering a sense of belonging and collective identity among teachers, which enhances their commitment to institutional goals (Kantos et al. 2023). These findings underscore the need for a deeper exploration of shared leadership’s impact on teachers’ organizational perceptions in centralized contexts.

The effectiveness of shared leadership varies across cultural and structural contexts, necessitating a comparative perspective. In decentralized systems, such as those in Finland and Australia, shared leadership thrives due to greater school autonomy and flexible governance, enabling teachers to take on leadership roles more readily (Harris and Jones 2021; Liu et al. 2021). In contrast, centralized systems like Türkiye’s impose constraints on teacher autonomy, yet shared leadership can still foster collaboration and empowerment within these limits (Özdemir et al. 2025). For example, Liu et al. (2021) found that shared leadership in Chinese schools, which are similarly centralized, enhances teacher motivation but requires strong principal support to overcome bureaucratic barriers. These cross-cultural comparisons highlight the universal principles of shared leadership—collaboration, trust, and participation—while underscoring the need to adapt its implementation to system-specific constraints (Bellibas et al. 2025). This study contributes to this discourse by examining shared leadership’s effects in Türkiye, offering insights into its applicability in a highly centralized context.

Despite its growing prominence, the literature on shared leadership in education remains limited, particularly in non-Western contexts. While foundational studies (e.g., Harris 2004; Pearce and Conger 2003) established its theoretical underpinnings, recent research highlights the need to examine its effects on specific outcomes like organizational attractiveness and identification, especially in hierarchical systems (Katıtaş et al. 2025; Paganin et al. 2023). This study addresses this gap by investigating how shared leadership influences teachers’ perceptions in Türkiye, contributing to both theoretical and practical discussions on educational leadership.

In analyzing the impact of leadership practices on teachers’ organizational perceptions in educational institutions, the concept of organizational attractiveness holds particular significance. Organizational attractiveness refers to the degree to which individuals perceive an organization as a positive, prestigious, and desirable workplace (Highhouse et al. 2003; Atalay et al. 2019). This perception directly affects employees’ attitudes toward the organization, their intention to stay, and their willingness to contribute voluntarily. In the educational context, teachers’ perception of their schools as attractive is associated with increased professional commitment, supports teaching quality, and indirectly contributes to student achievement (Akman and Özdemir 2018).

Organizational attractiveness is shaped by multiple factors, including physical working conditions, professional development opportunities, leadership style, institutional values, and overall school climate (Lievens et al. 2007). Recent studies emphasize additional influences such as workload, career progression, organizational support, and authentic leadership, which are associated with teachers’ perceptions of their workplace (Dotta et al. 2025). Positive perceptions of these elements not only are related to satisfaction and commitment but also are associated with teachers’ emotional and professional attachment to the institution.

These perceptions are closely linked to organizational identification. When teachers view their schools positively, they are more likely to internalize organizational goals, feel a sense of belonging, and align their professional identity with institutional values (Alev 2021; Kantos et al. 2023). Moreover, inclusive, and participatory leadership practices can be associated with organizational attractiveness and teachers’ identification, promoting greater engagement and motivation (Akman 2025). This interplay highlights the importance of improving school environments to support both organizational appeal and teacher commitment.

Organizational identification refers to individuals’ perception of themselves as part of the organization, experiencing an intrinsic unity with the organization’s goals and internalizing the corporate identity (Mael and Ashforth 1992). This concept is grounded in Social Identity Theory, which posits that individuals’ self-concepts are constructed through relationships with social groups and organizations to which they belong (Ashforth and Mael 1989). Teachers with prominent levels of identification contribute more to their schools’ objectives, assume voluntary responsibilities, and maintain high professional motivation (Ashforth et al. 2008; Riketta 2005; Özsarı and Kara 2024).

Within this framework, investigating the associations of shared leadership with teachers’ perceptions of organizational attractiveness and levels of organizational identification is meaningful not only theoretically but also in practical terms for educational management. Existing literature reports positive associations of shared leadership on both organizational attractiveness and organizational identification (Carson et al. 2007; Harris 2004; Katıtaş et al. 2025). However, studies evaluating these relationships using advanced statistical methods in education contexts—particularly in countries like Türkiye with centralized management structure remain limited. Hence, employing structural equation modeling (SEM), which enables testing complex relationships, can provide significant contributions to the field (Kline 2010).

This study was conducted with 381 teachers working in Malatya, Türkiye during the 2023–2024 academic year. The primary aim was to analyze the associations among teachers’ perceptions of shared leadership, organizational attractiveness, and organizational identification, and to examine these relationships confirmatively using SEM. Validated and reliable instruments employed for data collection included the Shared Leadership Scale (Özer and Beycioğlu 2013), Organizational Attractiveness Scale (Akman and Özdemir 2018), and Organizational Identification Scale (Mael and Ashforth 1992; adapted by Tak and Aydemir 2004).

Research Questions

To guide this investigation, the study addresses the following research questions:

1. What are the relationships among teachers’ perceptions of shared leadership, organizational attractiveness, and organizational identification in the context of Türkiye’s centralized education system?
2. To what extent do shared leadership and organizational identification predict teachers’ perceptions of organizational attractiveness?
3. How do contextual factors (e.g., educational level, teacher tenure) and additional constructs (e.g., school climate, psychological safety) influence the relationships among shared leadership, organizational attractiveness, and organizational identification?

The results of this research are expected to contribute to developing managerial strategies aimed at promoting teacher commitment, transforming school administrators’ leadership approaches, and constructing education policies based on participatory and collaborative structures. Thus, this study aims to make an original contribution to the literature by empirically examining the multidimensional associations of the shared leadership model with teachers’ organizational perceptions and attitudes within the Turkish context.

## 2. Method

### 2.1. Research Design

This study was conducted using a relational research design to investigate the relationships associations among teachers’ levels of shared leadership, organizational attractiveness, and organizational identification. Relational research seeks to explore associations between two or more variables and to provide insights into potential causal relationships (Büyüköztürk et al. 2013; Creswell 2012). To elucidate predictive relationships among the variables, Structural Equation Modeling (SEM) was employed, a statistical method widely utilized in relational research for its capacity to simultaneously analyze predictive relationships (Fraenkel et al. 2012).

### 2.2. Population and Sample

The study population comprised 381 teachers employed across various educational levels in Malatya, Türkiye, during the 2023–2024 academic year, who voluntarily participated in the research. Data were collected using three validated instruments: the Shared Leadership Scale developed by Özer and Beycioğlu (2013), the Organizational Attractiveness Scale developed by Akman and Özdemir (2018), and the Organizational Identification Scale originally developed by Mael and Ashforth (1992) and adapted into Turkish by Tak and Aydemir (2004).

This study included (Table 1) a total of 381 teachers from different school types in Malatya, including preschool, primary, middle, and high schools. Teachers were selected using stratified random samplings to ensure representation across seniority levels, gender, and educational levels (Creswell and Creswell 2018). To implement stratified random sampling, a sampling frame was obtained from the Malatya Provincial Directorate of Education, listing all teachers across public schools in Malatya. Strata were defined based on three variables: school type (preschool, primary, middle, high school), gender (male, female), and seniority (1–5 years, 6–10 years, 11–15 years, 16+ years). Proportional allocation was used to determine the number of teachers sampled from each stratum, reflecting the distribution of these characteristics in the population (e.g., approximately 40% middle school teachers, 50% male). Within each stratum, teachers were randomly selected using a random number generator to ensure unbiased selection. This approach ensured representativeness across key demographic and professional characteristics, enhancing the validity of the findings. The sample distribution was as follows: 50.7% male and 49.3% female; in terms of seniority, 12.6% had 1–5 years, 24.9% had 6–10 years, 24.9% had 11–15 years, and 37.5% had 16 years or more of teaching experience. Additionally, the duration of collaboration with their current principals varied (less than 1 year: 23.6%; 1–2 years: 27.0%; 2–3 years: 10.8%; 3–4 years: 17.6%; 5 years and above: 21.0%). Regarding educational level, the sample included preschool (5.0%), primary (37.5%), middle (39.1%), and high school (18.4%) teachers.

This distribution, covering teachers with varying experience, gender, educational level, and duration of collaboration with their principal, enhances the heterogeneity and representativeness of the sample. Although the sample does not fully represent all teachers in Malatya, the inclusion of teachers from different school types and seniority levels contributes to the generalizability of the findings. This limitation is explicitly discussed in the Section 7, and future research with larger and more representative samples is recommended (Leithwood et al. 2020; Özdemir et al. 2025).

### 2.3. Data Collection Procedure

In this study, data were collected using an online survey administered individually to each participating teacher. The survey link was sent directly to the teachers, who provided their responses after receiving detailed information regarding the purpose of the study, confidentiality measures, and the voluntary nature of participation. This procedure ensured that each participant could respond independently, maintaining both the reliability of the data and the ethical standards of the research.

### 2.4. Data Collection Instruments

The study employed a four-part battery test. The first part collected demographic information, while the second, third, and fourth parts consisted of the Shared Leadership Scale, Organizational Attractiveness Scale, and Organizational Identification Scale, respectively. Details of each scale are provided below.

*Shared Leadership Scale:* The study utilized the unidimensional, 10-item Shared Leadership Scale developed by Özer and Beycioğlu (2013). Confirmatory Factor Analysis (CFA) results for this study indicated acceptable fit indices: χ^2^ = 12, df = 33, χ^2^/df = 3.912, GFI = 0.93, AGFI = 0.88, NFI = 0.97, NNFI/TLI = 0.96, IFI = 0.97, CFI = 0.97, RMSEA = 0.088, RMR = 0.032, SRMR = 0.030. Cronbach’s Alpha internal consistency coefficient for this application was 0.96.

*Organizational Attractiveness Scale:* The Organizational Attractiveness Scale, developed by Akman and Özdemir (2018), was used. This unidimensional, 11-item scale’s structure was validated using second-order CFA, with fit indices consistent with established standards: χ^2^ = 202, df = 42, χ^2^/df = 4.82, GFI = 0.91, AGFI = 0.87, NFI = 0.94, NNFI/TLI = 0.94, IFI = 0.95, CFI = 0.95, RMSEA = 0.100, RMR = 0.044, SRMR = 0.033. Cronbach’s Alpha coefficient for this application was 0.95.

*Organizational Identification Scale:* The Organizational Identification Scale, originally developed by Mael and Ashforth (1992) and adapted into Turkish by Tak and Aydemir (2004), was employed. This unidimensional, 6-item scale was subjected to second-order CFA, yielding acceptable fit indices: χ^2^ = 32.7, df = 9, χ^2^/df = 3.63, GFI = 0.97, AGFI = 0.93, NFI = 0.96, NNFI/TLI = 0.95, IFI = 0.97, CFI = 0.97, RMSEA = 0.083, RMR = 0.029, SRMR = 0.031. The Cronbach’s Alpha coefficient for this application was 0.85.

### 2.5. Data Analysis

Data analysis was conducted using IBM SPSS V24.0 and IBM AMOS V24.0. Initially, the dataset was screened for errors, and incorrect entries were corrected based on the original forms. Subsequently, missing data were identified, with five forms containing missing values removed from the dataset. Outlier analysis was performed, resulting in the exclusion of seven participants’ data due to extreme values, leaving 389 observations. Eight additional participants’ data were excluded because they did not meet the univariate normality criteria, meaning their skewness and/or kurtosis values fell outside the acceptable range of −1 to +1, and/or their Z-scores exceeded ±3, which could distort parameter estimates and model fit in SEM (Çokluk et al. 2010). The final sample consisted of 381 participants.

For SEM, univariate and multivariate normality assumptions were evaluated. Univariate normality was assessed by ensuring skewness and kurtosis values fell between −1 and +1, and Z-scores ranged between −3 and +3 (Çokluk et al. 2010). Multivariate normality was evaluated, with results presented in Table 2. SEM was selected as the analytical technique because it allows for testing complex relationships among multiple variables simultaneously and enables the evaluation of both measurement and structural models concurrently (Kline 2010). This feature facilitated a comprehensive and confirmatory examination of the effects of teachers’ perceptions of shared leadership on organizational attractiveness and organizational identification.

The multivariate normality results (Table 2) indicate that the dataset satisfies multivariate normality assumptions (multivariate kurtosis = 0.359, critical ratio = 0.659). The analysis adopted criteria requiring multivariate kurtosis values to fall between −2 and +2 and a critical ratio less than zero (Bayram 2010). A two-stage approach was used for SEM, with Çelik and Yılmaz (2013) suggesting that measurement and structural models can be analyzed separately, with the first stage involving Confirmatory Factor Analysis (CFA). Accordingly, CFA was conducted as the first stage, with results reported in the Section 2.4. Given the dataset’s multivariate normality, the Maximum Likelihood estimation method, commonly used in such cases (Kline 2010), was selected, and Path Analysis, a standard SEM technique, was employed to evaluate the model.

## 3. Findings

This study examined the associations among teachers shared leadership, organizational attractiveness, and organizational identification levels using SEM. The theoretically grounded model was validated, with goodness-of-fit indices providing evidence of an acceptable model. The results revealed:

- A positive, moderate association between shared leadership and organizational attractiveness.
- A positive, moderate association between shared leadership and organizational identification.
- A positive, moderate association between organizational attractiveness and organizational identification.

Descriptive analysis results and inter-variable correlations are presented in Table 3.

As shown in Table 3, significant positive moderate correlations were found between shared leadership and organizational attractiveness (r = 0.656, *p* < .05), shared leadership and organizational identification (r = 0.538, *p* < .05), and organizational attractiveness and organizational identification (r = 0.583, *p* < .05). Standard deviations ranged from 4.45 to 10.25. Correlation coefficients were interpreted as weak (below 0.30), moderate (0.30 to 0.70), or strong (above 0.70) in absolute value (Cronk 2008).

### Model Analysis Results

**Figure 1:**
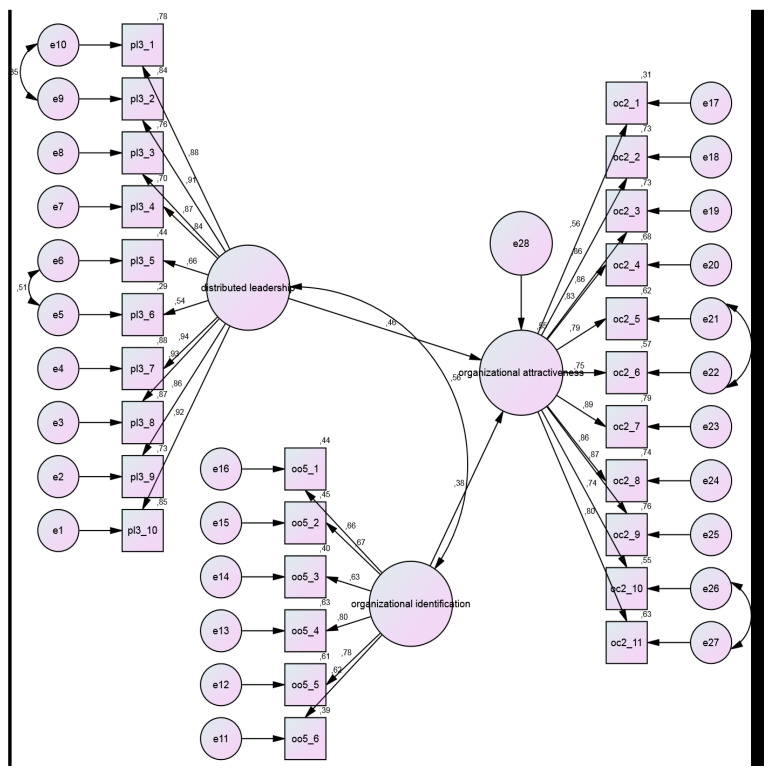
Path Diagram of the Model, Standardized Path Coefficients (Regression Coefficients), and Determination Coefficients (R^2^).

Standardized regression coefficients and their significance for the theoretically derived model (Figure 1) are presented in Table 4.

Table 4 indicates that all paths in the model were statistically significant (*p* < .01), supporting the validity of the proposed model. The goodness-of-fit indices, a key criterion for model acceptance (Byrne 2010; Kline 2010), are presented in Table 5.

Table 5 shows that the SEM model’s fit indices, including χ^2^/df (2.405), GFI (0.86), AGFI (0.84), NFI (0.92), NNFI/TLI (0.94), CFI (0.95), RMSEA (0.061), and SRMR (0.054), achieved “acceptable fit,” while IFI (0.95) indicated “good fit.” The validated model is depicted in Figure 1.

The SEM model (Figure 1) identifies organizational attractiveness (OA) as the primary dependent (endogenous) variable, directly influenced by the other variables. The validated model shows that organizational identification (OI) positively and directly predicts organizational attractiveness (β = 0.385, t = 6.052, *p* < .001), and shared leadership (SL) also positively and directly predicts organizational attractiveness (β = 0.457, t = 7.665, *p* < .001). Organizational identification accounts for approximately 15% of the variance in organizational attractiveness, while shared leadership explains about 21%. Collectively, organizational identification and shared leadership account for 55% of the variance in organizational attractiveness (R^2^ = 0.554).

## 4. Discussion

This study investigated the relationships among teachers’ perceptions of shared leadership, organizational attractiveness, and organizational identification in Türkiye’s centralized education system, using structural equation modeling (SEM). Grounded in Shared Leadership Theory (Pearce and Conger 2003; Carson et al. 2007), which emphasizes collective and participatory leadership, and Social Identity Theory (Ashforth and Mael 1989), which highlights group membership’s role in shaping identity, the findings provide significant insights into how these constructs interact. The results revealed moderate, statistically significant positive correlations among shared leadership and organizational attractiveness (r = 0.656, *p* < .05), shared leadership and organizational identification (r = 0.538, *p* < .05), and organizational attractiveness and organizational identification (r = 0.583, *p* < .05). SEM analysis further showed that shared leadership (β = 0.457, *p* < .001) and organizational identification (β = 0.385, *p* < .001) predict organizational attractiveness, explaining 55% of its variance (R^2^ = 0.554). These findings suggest that shared leadership fosters positive organizational perceptions and strengthens teachers’ institutional belonging, though the moderate correlations indicate contextual and psychological factors may influence these relationships (Hulpia and Devos 2010; Tschannen-Moran and Hoy 2000).

**Addressing the Research Questions**

The study was guided by three research questions, answered below using empirical findings, theoretical frameworks, and the context of Türkiye’s education system.

1. What are the relationships among teachers’ perceptions of shared leadership, organizational attractiveness, and organizational identification in the context of Türkiye’s centralized education system?

The findings (Table 3) confirmed moderate positive correlations among shared leadership, organizational attractiveness (r = 0.656, *p* < .05), and organizational identification (r = 0.538, *p* < .05), with organizational attractiveness and identification also correlated (r = 0.583, *p* < .05). These results suggest that shared leadership practices, such as collaborative decision-making, are associated with teachers viewing their schools as desirable workplaces and feeling greater institutional belonging. These associations align with Shared Leadership Theory’s emphasis on trust and collaboration (Pearce and Conger 2003) and Social Identity Theory’s focus on group-based identity (Ashforth and Mael 1989). However, the moderate strength of these correlations likely reflects constraints of Türkiye’s centralized education system, where limited school autonomy and high teacher workloads (24–30 teaching hours per week plus administrative duties) may restrict shared leadership’s impact (OECD 2023; Liu et al. 2021).

2. To what extent do shared leadership and organizational identification predict teachers’ perceptions of organizational attractiveness?

SEM results (Table 4) demonstrated that shared leadership (β = 0.457, *p* < .001) and organizational identification (β = 0.385, *p* < .001) significantly predict organizational attractiveness, accounting for 55% of its variance (R^2^ = 0.554, Figure 1). Shared leadership’s stronger effect (β = 0.457) underscores its role in fostering perceptions of schools as attractive workplaces, while organizational identification’s contribution (β = 0.385) highlights the importance of teachers internalizing institutional values. These findings support Shared Leadership Theory’s claim that distributed leadership enhances organizational outcomes (Carson et al. 2007) and Social Identity Theory’s assertion that identification drives positive perceptions (Riketta 2005). The model’s explanatory power emphasizes the practical value of promoting shared leadership and identification, even in a centralized system with limited teacher autonomy.

3. How do contextual factors (e.g., educational level, teacher tenure) and additional constructs (e.g., school climate, psychological safety) influence the relationships among shared leadership, organizational attractiveness, and organizational identification?

Subgroup analyses (Table 6) showed that middle school teachers (r = 0.68, *p* < .05) and those with 6–10 years of experience (r = 0.68, *p* < .05) reported stronger associations between shared leadership and organizational attractiveness compared to high school teachers (r = 0.60, *p* < .05) or those with over 16 years of experience (r = 0.55, *p* < .05). These differences may stem from variations in organizational culture or leadership opportunities across school types and career stages (Kantos et al. 2023). Unexamined constructs, such as school climate and psychological safety, likely mediate or moderate these relationships. For instance, a supportive climate may strengthen shared leadership’s impact by fostering trust (Hulpia and Devos 2010), while psychological safety could encourage leadership engagement (Tschannen-Moran and Hoy 2000). Additionally, emotional contagion among teachers may amplify shared leadership’s effects on identification, as shown by Paganin et al. (2023). The absence of these constructs in the model may explain the moderate correlations, suggesting future research should explore these mechanisms.

The moderate correlations (r = 0.538 to 0.656) likely reflect Türkiye’s centralized education system, where bureaucratic barriers and high workloads limit shared leadership’s implementation (Liu et al. 2021). Individual differences, such as tenure and gender, may also influence perceptions. For example, longer-tenured teachers may be less responsive to shared leadership due to entrenched hierarchical norms, while cultural gender dynamics could affect identification (Alev 2021). These findings align with international research, such as Hulpia et al. (2011), who linked shared leadership to organizational commitment, and Xu and Pang (2024), who found authentic leadership enhances commitment via identification. Unlike decentralized systems (e.g., Finland), where greater autonomy may amplify shared leadership’s effects (Harris and Jones 2021), Türkiye’s hierarchical context makes these associations noteworthy (Özer and Beycioğlu 2013).

The association between organizational attractiveness and identification aligns with Highhouse et al. (2003) and Akman and Özdemir (2018), who found that positive workplace perceptions foster institutional loyalty. However, contextual factors like school type and workload may moderate this relationship, with primary schools potentially fostering stronger attractiveness due to smaller class sizes (Kantos et al. 2023). In conclusion, this study demonstrates that shared leadership is associated with enhanced organizational attractiveness and identification, offering a strategic approach to improve teacher commitment in Türkiye’s centralized system. School leaders should adopt participatory practices, and policymakers should support reforms enabling shared leadership. Future research should incorporate longitudinal designs and additional constructs (e.g., psychological safety) to deepen understanding of these dynamics.

Additional constructs, such as school climate, psychological safety, or emotional contagion, may mediate or moderate these relationships. For example, a supportive school climate could strengthen the link between shared leadership and organizational attractiveness by fostering trust and collaboration, while psychological safety may enhance teachers’ willingness to engage in shared leadership roles (Hulpia and Devos 2010; Tschannen-Moran and Hoy 2000). Similarly, Paganin et al. (2023) found that emotional contagion among teachers mediates the relationship between leadership practices and team cohesion, suggesting that shared leadership may amplify positive emotional dynamics, further enhancing organizational identification. The absence of these variables in the current model may explain the moderate correlations, as they could account for additional variance in organizational outcomes. Future research should incorporate these constructs to provide a more comprehensive understanding of the mechanisms driving these relationships.

These findings are consistent with previous international research. For instance, Hulpia et al. (2011) found that sharing leadership responsibilities in school settings is associated with teachers’ organizational commitment and contributes to a positive organizational climate. Similarly, Carson et al. (2007) emphasized that shared leadership structures are related to an environment of trust and mutual support among team members, thereby associated with increased organizational attractiveness. More recent studies, such as Katıtaş et al. (2025), Liu et al. (2021), and Xu and Pang (2024), further confirm that shared leadership enhances teacher self-efficacy, job satisfaction, and organizational commitment across diverse contexts, including centralized systems like China and Türkiye. These studies support the current findings regarding the positive associations among shared leadership, organizational attractiveness, and identification.

However, Harris (2004) cautioned that the associations of shared leadership cannot be evaluated independently of context. The structural and cultural characteristics of education systems significantly influence the effectiveness of leadership models. Given the centralized and hierarchical nature of Türkiye’s education system, the current findings are particularly noteworthy, as they demonstrate that shared leadership can be positively associated with teacher perceptions even within such rigid institutional structures. In contrast, in decentralized systems like Finland, shared leadership may yield stronger effects due to greater teacher autonomy (Harris and Jones 2021). In this regard, the present study makes an original contribution to the literature concerning the applicability of the model within the Turkish context (see Özer and Beycioğlu 2013; Özdemir et al. 2025).

The positive association found between organizational attractiveness and organizational identification in this study is also consistent with the findings of Highhouse et al. (2003), as well as Lievens et al. (2007). These studies emphasized that perceiving an organization as positive, reputable, and desirable to work is associated with individuals’ institutional loyalty and organizational identification. Similarly, Akman and Özdemir (2018) found that teachers who perceive their schools as attractive are more likely to identify with the institutional identity and show higher levels of professional commitment.

Nevertheless, the moderate strength of this association in the present study suggests that it may be influenced by contextual variables such as school type, leadership style, teacher–student relations, and organizational culture. For example, primary school teachers may perceive higher organizational attractiveness due to smaller class sizes and stronger community ties, while high school teachers may face greater workload pressures, weakening their identification (Kantos et al. 2023). Furthermore, since perceptions of organizational attractiveness are inherently subjective, individual differences (e.g., age, tenure, subject area, years of service) and the socio-cultural context of schools should also be taken into consideration.

In conclusion, this study highlights the potential associations of shared leadership with positive organizational perceptions and a stronger sense of institutional belonging among teachers. In this regard, promoting more participatory, collaborative, and trust-based leadership practices in educational institutions may be considered a strategic approach to supporting teachers’ levels of organizational attractiveness and identification. The findings offer valuable insights for school leaders in reconsidering their leadership practices, and for policymakers aiming to develop reforms that strengthen teacher commitment.

## 5. Theoretical Contribution

This study offers significant contributions to two key theoretical frameworks—Social Identity Theory (Ashforth and Mael 1989) and Shared Leadership Theory (Spillane 2006)—by examining the associations among teachers’ perceptions of shared leadership, organizational attractiveness, and organizational identification.

Social Identity Theory focuses on the processes through which individuals identify with the groups or organizations to which they belong. According to this theory, when individuals perceive themselves as members of an organization, they internalize their values and norms, which in turn shapes their organizational commitment and behavioral tendencies (Ashforth et al. 2008; Tajfel and Turner 1986). The findings of this study indicate that shared leadership practices are associated with teachers’ levels of organizational identification, which positively predicts their perceptions of organizational attractiveness. This demonstrates that Social Identity Theory remains applicable in the context of educational organizations and that shared leadership may be related to the development of teachers’ institutional identity. These results align with Riketta’s (2005) meta-analytic findings, which suggest that organizational identification is associated with various outcomes such as job satisfaction, performance, and organizational commitment. Furthermore, Xu and Pang (2024) found that authentic leadership fosters organizational commitment through teachers’ identification, reinforcing the role of leadership in strengthening social identity processes in schools.

The study also contributes to Shared Leadership Theory at the theoretical level. Developed by Spillane (2006), this theory posits that leadership is not an individual act but a collective and interactive process. In this framework, leadership is seen as a dynamic structure shared among different actors within the organization through mutual interactions. The findings of the present study show that in environments where leadership roles are distributed among teachers, both their sense of institutional belonging and their positive evaluations of the organization are strengthened. These results suggest that shared leadership practices are potentially associated with not only improved administrative effectiveness but also support for teachers’ psychological needs and emotional engagement. Recent research, such as Katıtaş et al. (2025), Liu et al. (2021), and Paganin et al. (2023), further supports this, demonstrating that shared leadership enhances teacher self-efficacy, organizational commitment, and team cohesion across diverse contexts, including centralized systems like China and Türkiye. Specifically, Paganin et al. (2023) highlights that emotional contagion mediates the relationship between leadership practices and team outcomes, suggesting that shared leadership fosters positive emotional dynamics that amplify teachers’ identification and commitment. Pearce and Conger (2003) similarly argued that shared leadership is positively associated with psychological constructs such as trust, commitment, and self-efficacy.

These findings provide theoretical validation for the applicability of shared leadership in diverse educational systems. In centralized systems like Türkiye, where teacher autonomy is limited by hierarchical structures and high workloads (OECD 2023), shared leadership fosters collaboration within these constraints, as evidenced by the moderate correlations in this study (r = 0.538 to 0.656). In contrast, in decentralized systems like Finland or Australia, shared leadership may yield stronger effects due to greater flexibility and teacher empowerment (Harris and Jones 2021). These cross-cultural comparisons highlight the theory’s universal principles—collaboration, trust, and participation—while underscoring the need for context-specific adaptations. For instance, the psychological mechanisms underlying shared leadership, such as emotional contagion and psychological safety, may amplify its effects on organizational identification, as supported by Paganin et al. (2023) and Hulpia and Devos (2010). This study’s findings in Türkiye’s centralized context thus contribute to a broader understanding of Shared Leadership Theory’s cross-cultural applicability, extending its theoretical scope by demonstrating its relevance in a non-Western, hierarchical educational system (Cobanoğlu 2020).

In conclusion, this research strengthens the theoretical validity of Social Identity Theory by evaluating its organizational identification dimension in an educational context. At the same time, it contributes a cross-cultural perspective to the literature by offering new evidence on the applicability of Shared Leadership Theory. In this regard, the study provides a valuable contribution to theoretical discussions on the impact of leadership processes on individuals’ organizational attitudes and behaviors. Future research may explore the effects of different contextual variables (e.g., school type, administrative structure, teacher experience) on the relationships among leadership, identification, and attractiveness within these theoretical frameworks.

## 6. Practical Recommendations

The findings of this study reveal the associations among shared leadership, organizational attractiveness, and organizational identification within educational organizations, offering concrete and functional recommendations for educational administrators, policymakers, and practitioners. These recommendations have the potential to support both structural and cultural transformations in educational settings. Grounded in the study’s empirical findings, particularly the significant correlations (r = 0.538 to 0.656, *p* < .05) and the 55% variance in organizational attractiveness explained by shared leadership and organizational identification (R^2^ = 0.554), these recommendations provide actionable strategies. However, given Türkiye’s highly centralized education system, with limited school autonomy and high teacher workloads (Eurydice 2024; OECD 2023), their implementation may require tailored policy support and cultural adaptation, potentially limiting generalizability to decentralized systems.

1. **Promoting a Culture of Shared Leadership in Schools:** The research findings indicate that shared leadership practices are associated with teachers’ positive attitudes toward the organization and their levels of commitment. Accordingly, school administrators should adopt a democratic management approach that allows teachers to actively participate in decision-making processes rather than restricting leadership roles to a single individual. For example, establishing teacher-led committees to adapt curriculum components within MoNE guidelines or involving teachers in strategic planning for school improvement initiatives can enhance perceptions of organizational attractiveness and foster a sense of ownership (Harris 2004; Leithwood et al. 2020). Such participatory practices are likely to promote trust-based relationships within the school and encourage collaboration toward shared goals.
2. **Restructuring Professional Development Programs with a Leadership Perspective:** The study’s findings highlight that shared leadership is positively associated with organizational attractiveness and identification (β = 0.457, *p* < .001), suggesting that empowering teachers as leaders can enhance their engagement. Therefore, educational policies should be redesigned not only to enhance teachers’ instructional skills but also to develop their leadership capacities. Policymakers should design comprehensive in-service training programs, especially targeting school administrators and teacher leaders to integrate the principles and practices of shared leadership into the school environment. These programs could include workshops on collaborative decision-making, conflict resolution, and team management, tailored to address the specific needs of teachers in centralized systems (Özer and Beycioğlu 2013). By fostering teachers’ leadership skills, these programs can support the participatory culture shown to predict organizational attractiveness in this study. However, in Türkiye’s centralized system, where curriculum and training are tightly controlled by the Ministry of National Education, implementing such programs may require national policy reforms, such as MoNE guidelines granting schools limited autonomy to design teacher-led professional development initiatives.
3. **Improving Physical and Social Conditions of Schools:** The study demonstrates that organizational attractiveness, influenced by shared leadership and identification (R^2^ = 0.554), is critical to teachers’ commitment. Improving physical and social conditions is essential to creating an attractive workplace that supports these outcomes. Accordingly, it is important to organize school facilities functionally and aesthetically to meet teachers’ professional needs. For instance, providing modern classroom resources and comfortable staff rooms can enhance teachers’ workplace satisfaction. Moreover, establishing transparent and open communication channels within the institution is associated with trust and solidarity among teachers, thereby supporting institutional belonging. Implementing regular feedback forums where teachers can voice concerns to administrators or creating online platforms for collaborative planning can strengthen organizational attractiveness (Akman and Özdemir 2018). In Türkiye’s context, where resource disparities between urban and rural schools persist (OECD 2023), implementing these improvements may face challenges, requiring targeted investments and policy support to ensure equitable access to enhanced conditions.
4. **Establishing a Shared Vision and Values:** Organizational identification is closely linked to teachers’ internalization of the school’s mission and values. In this context, school culture should be structured in a way that teachers perceive themselves as integral parts of the school. Actively involving teachers in co-creating the school’s vision through workshops or focus groups will facilitate the adoption and internalization of these principles. A school culture built around shared values can create a meaningful sense of unity among teachers, thereby increasing levels of identification (Riketta 2005). For example, organizing annual vision-setting meetings where teachers contribute to defining institutional goals can strengthen their sense of belonging. Additionally, such a cultural structure can contribute to positive long-term outcomes, including teacher commitment, job satisfaction, and organizational citizenship behaviors.
5. **Contextual Analysis of Implementation Variations:** Finally, considering the contextual findings of the study, it is important to acknowledge that the implementation of shared leadership and organizational processes may vary depending on cultural, structural, and managerial conditions. In countries with centralized and hierarchical administrative systems, such as Türkiye, promoting these practices requires supportive regulations at the policy level as well as managing cultural change processes at the school level. For instance, MoNE could pilot shared leadership programs in select schools to evaluate their feasibility before nationwide implementation. Policies aligned with the overall educational system, yet incorporating transformational leadership approaches, can render shared leadership more sustainable.

## 7. Limitations

This study has revealed significant findings by examining the associations among teachers’ shared leadership, organizational attractiveness, and organizational identification levels; however, there are several limitations that should be considered when interpreting the results.

First, the sample is limited to teachers working in public schools located in Malatya province, in the Eastern Anatolia Region of Türkiye. This restricts the generalizability of the findings to the broader teacher population across Türkiye or to other provinces and regions with differing cultural, administrative, and socio-economic contexts. Given the heterogeneous nature of Türkiye’s education system, marked by regional inequalities and varying school types, future studies employing diverse samples could more robustly evaluate the contextual validity of the results.

Second, the use of self-report scales for data collection may have introduced social desirability bias, whereby participants tend to respond in a more favorable manner. This risk is especially salient for normative and positively valued constructs such as shared leadership, where participants might report idealized perceptions rather than their actual experiences. Additionally, common method bias (CMB) may have inflated the observed correlations (e.g., r = 0.656 for SL-OA) due to the reliance on self-report measures for all variables (Podsakoff et al. 2003). This could have overestimated the strength of relationships, potentially affecting the validity of the findings. Cultural norms in Türkiye, where teachers may feel pressured to report positive perceptions of leadership practices, could further exacerbate response bias. Consequently, this methodological issue may limit the accuracy and validity of the findings.

Third, this research employed a cross-sectional design, wherein data were collected at a single point in time. As a result, the findings reflect correlational relationships rather than causal effects, limiting the ability to draw definitive conclusions about the directionality and causality of the observed associations. For example, while shared leadership is positively associated with organizational attractiveness, longitudinal research is needed to establish potential causal mechanisms underlying this relationship.

Fourth, the study’s model, by focusing exclusively on shared leadership, organizational attractiveness, and organizational identification, provides only a partial understanding of the complex processes influencing teachers’ organizational perceptions. Additional constructs, such as school climate, teacher motivation, leader-member exchange, or psychological safety, as noted earlier, could mediate or moderate these relationships, offering a more comprehensive model (Hulpia and Devos 2010; Tschannen-Moran and Hoy 2000). This narrow scope limits the explanatory power of the current model and underscores the need for broader frameworks in future research.

In sum, these limitations suggest cautious interpretation of the findings. Future research enriched by larger and more representative samples, diverse data collection methods, broader theoretical models, and longitudinal designs would enhance the generalizability and theoretical validity of the results.

## 8. Future Research

This study contributes substantially to educational management literature by elucidating the associations among shared leadership, organizational attractiveness, and organizational identification. Nonetheless, the findings open avenues for extension and deeper inquiry into future investigations.

First, as the current data were collected from a specific region in Türkiye, it is recommended to replicate similar studies across different geographic areas, schools with varying socio-economic profiles, and educational levels (elementary, middle, and high school) to test the generalizability of the findings in broader contexts. Given the heterogeneous nature of Türkiye’s education system—characterized by a centralized hierarchy under the Ministry of National Education (MoNE), limited school autonomy (e.g., restricted budget and curriculum adaptation powers), and high teacher workloads (often exceeding 40 h weekly including extracurricular responsibilities)—future studies employing diverse samples could more robustly evaluate the contextual validity of the results (Eurydice 2024; OECD 2023). For instance, while our sample from Malatya reflects urban-rural divides, comparative research in more autonomous private institutions or regions with varying centralization levels (e.g., Istanbul vs. Eastern Anatolia) would test transferability. Internationally, these findings may inform systems with similar centralization challenges, such as those in other OECD countries transitioning toward decentralized models, by highlighting shared leadership’s association with mitigating workload-related burnout and promoting teacher commitment. Such research would enable assessment of whether the shared leadership model yields comparable organizational outcomes across diverse demographic and institutional settings.

Second, to strengthen causal explanations regarding the associations of shared leadership with organizational attractiveness and identification, the application of longitudinal research designs is necessary. These studies would track changes in leadership practices and teachers’ organizational perceptions over time, allowing for more robust testing of potential causal relationships. Incorporating potential mediators and moderators—such as school climate, teacher motivation, leader-member exchange, or psychological safety—into the model would permit the examination of more intricate and explanatory theoretical frameworks. For example, school climate may mediate the relationship between shared leadership and organizational attractiveness, while psychological safety could moderate the impact of leadership practices on identification, providing a more comprehensive understanding of these dynamics (Hulpia and Devos 2010; Tschannen-Moran and Hoy 2000).

Third, given the quantitative nature of this study, there is a need for qualitative or mixed-methods research to deepen understanding and capture teachers’ lived experiences more holistically. Methods such as interviews, focus group discussions, and observations could provide multifaceted insights into how teachers perceive shared leadership practices and how these are associated with their motivation, identification, and organizational commitment.

Finally, considering the impact of cultural contexts on leadership perceptions and practices, testing similar models in different countries is crucial to delineate the universal versus context-dependent aspects of shared leadership. Comparative international studies conducted in countries with varying centralized and decentralized educational systems would offer valuable opportunities to evaluate the effectiveness and cross-cultural validity of shared leadership. In this regard, examining Spillane’s (2006) shared leadership theory beyond Western contexts, within countries characterized by diverse governance structures and cultural value systems, could yield more comprehensive conclusions regarding the theory’s universal applicability.

In conclusion, future research should adopt multidimensional, interdisciplinary, and cross-cultural perspectives to better understand the associations of shared leadership with teachers and to facilitate its effective integration into educational systems.

## 9. Conclusions

This study examined the associations among shared leadership, organizational attractiveness, and organizational identification levels of 381 teachers working in Türkiye using structural equation modeling (SEM). The results indicated positive and moderate significant correlations between shared leadership and organizational attractiveness (r = 0.656, *p* < .05), shared leadership and organizational identification (r = 0.538, *p* < .05), and organizational attractiveness and organizational identification (r = 0.583, *p* < .05). Moreover, SEM analysis demonstrated that shared leadership and organizational identification significantly predicted organizational attractiveness, together explaining 55% of the variance in organizational attractiveness (R^2^ = 0.554). These findings highlight the strong association of the shared leadership model with teachers’ organizational perceptions and offer meaningful theoretical and practical implications for the field of educational management.

The study provides a unique contribution to the literature, given its context within the highly centralized and hierarchical Turkish education system. By demonstrating how shared leadership is associated with teachers’ psychological bonds and perceptual processes with their organizations, the research offers important theoretical support for Social Identity Theory (Ashforth and Mael 1989) alongside Spillane’s (2006) shared leadership framework in educational contexts. Notably, the positive associations found between teachers’ organizational attractiveness, identification, and shared leadership suggest that distributing leadership roles through participatory mechanisms is related to teachers’ sense of belonging and positively influences their organizational attitudes.

The findings provide strategic guidance for practitioners and policymakers. School administrators are encouraged to share leadership roles with teachers, promoting their active involvement in decision-making processes and positively influencing organizational perceptions. This approach is associated with organizational attractiveness and identification and promotes stronger alignment with institutional goals, higher job satisfaction, and improved performance among teachers. Education policymakers should prioritize professional development programs that empower school leaders to implement shared leadership effectively. Simultaneously, policies aimed at improving physical and social conditions shaping teachers’ organizational perceptions—such as transparent communication channels, participatory school climates, and shared values—should be emphasized to reinforce teachers’ emotional attachment to their institutions.

This study underscores that shared leadership is not merely an administrative model but also a psychological process supporting teachers’ organizational commitment and identity construction. Consequently, shared leadership can be strategically utilized in educational transformation processes to positively influence teachers’ institutional experiences. Offering a distinctive perspective on a relatively underexplored topic within the Turkish context, this research also lays the groundwork for future studies in diverse cultural and structural environments.

In summary, this study supports the functional role of leadership approaches in education at the theoretical level and proposes concrete strategies for improving teachers’ organizational attitudes at the applied level. Future research should investigate potential mediating and moderating variables to deepen understanding of this model. Additionally, longitudinal studies supported by qualitative methods can reveal the temporal associations of shared leadership practices, facilitating the development of comprehensive and sustainable policy recommendations in educational management.

## Figures and Tables

**Table 1:** Demographic Characteristics of Study Population and Sample.

Variable	Category	n	%
Gender	Male	193	50.7
	Female	188	49.3
	Total	381	100.0
Seniority	1–5 years	48	12.6
	6–10 years	95	24.9
	11–15 years	95	24.9
	16 years and above	143	37.5
	Total	381	100.0
Duration Collaborating with Current Principal	Less than 1 year	90	23.6
	1–2 years	103	27.0
	2–3 years	41	10.8
	3–4 years	67	17.6
	5 years and above	80	21.0
	Total	381	100.0
Educational Level	Preschool	19	5.0
	Primary School	143	37.5
	Middle School	149	39.1
	High School	70	18.4
	Total	381	100.0

**Table 2:** Multivariate Normality Analysis.

Variable	Skewness	Critical Ratio (CR)	Kurtosis	Critical Ratio (CR)
Shared Leadership	−0.678	−5.404	−0.158	−0.629
Organizational Attractiveness	−0.253	−2.018	−0.484	−1.927
Organizational Identification	−0.371	−2.960	−0.358	−1.426
Multivariate			0.359	0.639

**Table 3:** Descriptive Analysis Results and Inter-Variable Correlation Coefficients.

	$\bar{x}$	ss	min	max	1. SL	2. OI
1. SL	37.34	9.37	11	50	1.00	
2. OI	22.96	4.45	12	30	0.538	1.00
3. OA	36.49	10.25	11	55	0.656	0.583

Note: SL = Shared Leadership, OI = Organizational Identification, OA = Organizational Attractiveness, *p* < .01.

**Table 4:** Model Analysis Results.

Dependent Variable	Independent Variable	(Unstandardized Coefficient) B	(Standardized Coefficient) β	S.E. (Standard Error)	C.R. (Critical Ratio/t)	*p*
Organizational Attractiveness (OA)	Shared Leadership (SL)	0.318	0.457	0.042	7.665	***
Organizational Attractiveness (OA)	Organizational Identification (OI)	0.527	0.385	0.087	6.052	***

Notes: χ^2^ = 762.488, df = 317, *p* < .01 (The overall model fit is significant). B: Unstandardized regression coefficient. β: Standardized regression coefficient. S.E.: Standard error. C.R.: Critical ratio (t-value). *p* Significance level (*** *p* < .001). Abbreviations: SL = Shared Leadership, OI = Organizational Identification, OA = Organizational Attractiveness.

**Table 5:** Goodness-of-Fit Indices for the Model.

Fit Index	Acceptable Fit	Good Fit	Obtained Values	Interpretation
χ^2^/sd	2 ≤ χ^2^/sd ≤ 5	0 ≤ χ^2^/sd < 2	2.405	Acceptable Fit
GFI	0.90 ≤ GFI < 0.95	0.95 ≤ GFI ≤ 1.00	0.86	Marginally Acceptable *
AGFI	0.85 ≤ AGFI < 0.90	0.90 ≤ AGFI ≤ 1.00	0.84	Marginally Acceptable *
NFI	0.90 ≤ NFI < 0.95	0.95 ≤ NFI ≤ 1.00	0.92	Acceptable Fit
NNFI/TLI	0.95 ≤ NNFI < 0.97	0.97 ≤ NNFI ≤ 1.00	0.94	Acceptable Fit
IFI	0.90 ≤ IFI < 0.95	0.95 ≤ IFI ≤ 1.00	0.95	Good Fit
CFI	0.95 ≤ CFI < 0.97	0.97 ≤ CFI ≤ 1.00	0.95	Acceptable Fit
RMSEA	0.05 ≤ RMSEA ≤ 0.08	0 ≤ RMSEA < 0.05	0.061	Acceptable Fit
RMR	0.05 ≤ RMR ≤ 0.08	0 ≤ RMR < 0.05	0.061	Acceptable Fit
SRMR	0.05 ≤ SRMR ≤ 0.08	0 ≤ SRMR < 0.05	0.054	Acceptable Fit

Sources: (Bayram 2010; Brown 2006; Çelik and Yılmaz 2013; Harrington 2009; Hu and Bentler 1999; Kline 2010; Schermelleh-Engel et al. 2003; Sümer 2000; Şimşek 2007). Notes: * Marginally Acceptable: GFI (0.86) and AGFI (0.84) are slightly below the conventional thresholds (GFI ≥ 0.90, AGFI ≥ 0.85) but are considered marginally acceptable in the context of complex SEM models with large sample sizes (Schermelleh-Engel et al. 2003). References: (Bayram 2010; Brown 2006; Çelik and Yılmaz 2013; Harrington 2009; Hu and Bentler 1999; Kline 2010; Schermelleh-Engel et al. 2003; Sümer 2000; Şimşek 2007). All other indices (χ^2^/sd, NFI, NNFI/TLI, IFI, CFI, RMSEA, RMR, SRMR) indicate acceptable or good fit, supporting the overall model adequacy.

**Table 6:** Subgroup Analysis of Correlations by Educational Level and Tenure.

Variable	Educational Level	SL-OA Correlation	Tenure	SL-OA Correlation
Preschool	0.62 **	1–5 years	0.64 **	
Primary	0.65 **	6–10 years	0.68 **	
Middle	0.68 **	11–15 years	0.63 **	
High School	0.60 **	16+ years	0.55 **	

Note: SL = Shared Leadership, OA = Organizational Attractiveness, *p* < .01. ** Shared leadership is a universal predictor of organizational attractiveness across all subgroups (*p* < .01), but its influence peaks among middle school teachers and mid-tenure professionals (6–10 years)—ideal targets for leadership development initiatives.

## Data Availability

The data utilized in this study is not publicly available due to confidentiality and privacy obligations. However, data access may be granted upon reasonable request to the corresponding author, subject to appropriate ethical approvals.
